# Novel dsRNA designs increase the stability and bioefficacy of RNAi-based pesticides for *Varroa destructor*

**DOI:** 10.3389/finsc.2026.1868457

**Published:** 2026-08-17

**Authors:** Biko K. Muita, James E. Damayo, Emily J. Remnant, Neil A. Smith, Ian K. Greaves, Amol B. Ghodke, Nagalingam Kumaran, John M. K. Roberts

**Affiliations:** 1 CSIRO, Health and Biosecurity Research Unit, Canberra, ACT, Australia; 2 School of Life and Environmental Sciences, The University of Sydney, Sydney, NSW, Australia; 3 CSIRO, Agriculture and Food Research Unit, Canberra, ACT, Australia; 4 CSIRO, Health and Biosecurity Research Unit, Brisbane, QLD, Australia

**Keywords:** honey bee, parasitic mites, double stranded RNA, RNA interference, miticide

## Abstract

*Varroa destructor* is a major global pest of the European honey bee, and RNA interference (RNAi) has emerged as a promising technology for the development of effective control tools. However, the efficacy of RNAi-based approaches remains highly variable, due in part to the rapid degradation and inefficient delivery of exogenously applied double stranded RNA (dsRNA) This study investigated whether advanced dsRNA molecular designs could improve dsRNA stability, processing, and biological outcomes in the honey bee-*Varroa* mite pathosystem. Loop-ended dsRNA (ledRNA) constructs incorporating either G-U wobble base-pairing or asymmetric bulge modifications were designed to target two *Varroa* genes: the chitin-binding protein *Peritrophin-A-like (Pero)* and the neuropeptide crustacean hyperglycemic hormone (*CHH*). Stability assays, immersion and indirect feeding bioassays, and small RNA sequencing were used to evaluate dsRNA persistence, RNAi activity, and biological efficacy compared to conventional dsRNA. The advanced designs exhibited double the stability in sucrose feeding solutions and within adult bees compared with conventional dsRNA. In immersion and indirect feeding bioassays, these designs produced variable but enhanced effects on target gene silencing (~36-86% reduction) and mite mortality (~32-56% increase) when compared with conventional dsRNA. Small RNA sequencing revealed effective processing of the advanced dsRNA designs, with complete siRNA coverage of the target region, 22-24 nt peak size classes and antisense strand bias. Together, these results demonstrate that advanced dsRNA molecules have increased stability, effective processing, and strong efficacy in *V. destructor* under laboratory conditions, supporting further investigation of structurally optimized dsRNA designs for field hive applications.

## 1 Introduction

Invertebrate pests are an ongoing biosecurity issue for global agriculture. Their management continues to be reliant on broad spectrum pesticides with unwanted off-target effects and impacts on the environment (1). The effectiveness of pesticides is also increasingly undermined by pests developing resistance. This has led to a growing demand for more sustainable pest treatments that support integrated pest management principles. Biopesticides based on RNA interference (RNAi) technology are a potential solution that has advanced over a decade. RNAi is a conserved gene-silencing mechanism where double stranded RNA (dsRNA) is processed into small interfering RNA (siRNA) that act to guide the degradation of complementary RNA (2). Designing targeted dsRNA to pest genes enables sequence-based species-specific disruption of target genes to induce mortality or reduce fitness of pests. While RNAi has been most successful with transgenic crop plants, where dsRNA is delivered endogenously (3–5), pest management through topical or oral delivery of dsRNA (exogenous RNAi) continues to be a challenge.

Exogenous delivery of dsRNA can have highly variable and often inconsistent efficacy across invertebrate taxa. Firstly, environmental factors that degrade dsRNA before it is ingested are a significant problem for field applications. Ultraviolet (UV) radiation is a dominant factor, inducing photochemical damage and strand breaks that can inactivate dsRNA within hours (6). Double stranded RNA is also highly susceptible to extracellular RNases produced by microorganisms in soil, water, and on leaves. Cuticular penetration and systemic movement of intact dsRNA are other factors that can limit delivery to target pests (7, 8). Once ingested, dsRNA faces further degradation by gut nucleases and extreme pH conditions (9, 10). Cellular uptake of intact dsRNA also presents a challenge, where most arthropods lack efficient and conserved mechanisms for trans-epithelial transport of dsRNA (11). Overcoming these internal and external environmental factors has been a major barrier to widespread application of RNAi-based biopesticides in agriculture.

Recent advances in RNAi technology have focused on improving the stability, mobility and biological processing of exogenous dsRNA to achieve more reliable gene silencing under field conditions. These advances include various nanoparticle carriers such as clay nanosheets, cell penetrating peptides and liposomal encapsulation, for improved delivery of RNAi-based formulations (12–14). Chemical modification of dsRNA has also been used to improve resistance to nucleases (15, 16). Other key approaches have used sequence modifications that alter the architecture of the dsRNA molecule. A foundational design is the hairpin dsRNA (hpRNA) where self-complimentary single-stranded RNA folds to produce a double-stranded stem-loop structure (15). This has since enabled development of other advanced designs for enhanced RNAi efficacy. For instance, loop-ended dsRNA (ledRNA) containing terminal loops on both ends of the dsRNA stem, has been shown to provide improved resistance to exonucleases and facilitate systemic movement and gene silencing in plants and insects (17, 18). The introduction of small mismatches in the sense strand of the dsRNA stem has also been used to improve dsRNA stability and enzymatic processing, such as the incorporation of G-U base pairs to reduce self-silencing (19–21) or the creation of asymmetric bulges with single nucleotide deletions to alter the size distribution of resulting siRNAs (22). These strategies offer substantial improvements for effective exogenous dsRNA applications, where complex barriers to dsRNA uptake and processing in invertebrate pests can limit RNAi efficacy.

Honey bees (*Apis mellifera*) provide a useful invertebrate system for examining exogenous dsRNA, as they are amenable to controlled oral delivery of dsRNA and have been the focus of extensive RNAi research (23–25). Importantly, dsRNA ingested by bees is shown to be transferred to parasitic mites during feeding, enabling host-mediated delivery to the target pest (23). The parasitic bee mite, *Varroa destructor*, is the most significant threat to global apiculture, with mites weakening colonies by feeding on developing brood and adult bees and transmitting multiple bee viruses (26–28). Previous studies have shown that dsRNA can suppress Varroa mites and other bee pests and pathogens, via direct immersion, injection and bee-mediated delivery, producing significant gene knockdown, reduced mite survival or impaired reproduction (23, 29–32). More recently, these effects have been demonstrated in semi-field and field conditions (33, 34), culminating in the release of a Varroa RNAi biopesticide, Norroa™, which targets a calmodulin-like gene to suppress mite reproduction (35). While this represents a major advancement for RNAi-based pest control in apiculture, this biopesticide does not induce mite mortality and relies on conventional dsRNA molecules. RNAi tools that can achieve rapid knockdown of mite outbreaks and have longer stability within the hive environment and honey bee host would be highly beneficial for managing Varroa mites.

To determine whether an improved molecular design can enhance RNAi performance against Varroa, we investigated the biological activity of several advanced dsRNA designs using the honey bee-Varroa pathosystem. We focused on a ledRNA architecture paired with two different sequence modifications (G-U base pairing and asymmetric bulge) and assessed their efficacy across two Varroa gene targets. The chitin-binding protein Peritrophin-A-like (Pero) is involved in maintaining the peritrophic membrane in the gut, while crustacean hyperglycemic hormone (CHH) is a neuropeptide that regulates energy metabolism, osmoregulation, and stress responses. These targets were selected based on previous studies that demonstrated that their silencing resulted in moderate levels of mite mortality, making them suitable candidates for evaluating whether advanced dsRNA designs could further improve RNAi efficacy (30, 37). Using direct contact and bee-mediated delivery of dsRNA, we demonstrated that these advanced molecules do provide improved RNAi efficacy, in terms of gene silencing and mite mortality. We also compared the siRNA profiles in treated Varroa mites to gain further insights on how the processing of these advanced molecules may influence performance of RNAi.

## 2 Materials and methods

### 2.1 Arthropod source and maintenance

*Varroa destructor* mites were collected from honey bee colonies maintained at the University of Sydney and Macquarie University. Mite collection occurred at least 30 days following treatment with Bayvarol^®^ or Apivar^®^ strips. Mites were obtained using two methods. First, brood frames were removed from hives and transported to the laboratory, where worker brood cells were uncapped and mites harvested with a fine paintbrush and placed into Petri dishes lined with filter paper. Second, adult bees were dusted with powdered sugar and gently agitated to dislodge dispersal mites (36), which were then transferred with a fine paintbrush to filter paper-lined Petri dishes. All collected mites were maintained at room temperature until use.

Honey bees were sourced from donor colonies maintained at the CSIRO Black Mountain laboratory. Brood frames were collected and kept at 35 °C to obtain pupae and newly emerged worker bees for mite immersion and bee-feeding experiments, respectively.

### 2.2 dsRNA preparation using *in vitro* transcription

Two target genes Pero and CHH for *V. destructor* were selected from previous studies (30, 37). Both genes were shown to be effectively silenced using conventional dsRNA and cause moderate (30-50%) mite mortality. Gene-specific primers incorporating the T7 promoter sequence (5′-TAATACGACTCACTATAGGG-3′) were designed using Primer3 software (38). All primer sequences are listed in **Supplementary Table 1**.

Target gene fragments of 500 bp for the conventional dsRNA were amplified via PCR using Q5^®^ High-Fidelity DNA Polymerase (NEB) under the following cycling conditions: initial denaturation at 98 °C for 30 s, followed by 15 cycles of 98 °C for 10 s, 60-64 °C for 20 s, and 72 °C for 30 s, then 25 cycles of 98 °C for 10 s, 70-72 °C for 20 s, and 72 °C for 30 s, with a final extension at 72 °C for 2 min. PCR amplicons were purified using a QIAquick PCR Purification Kit (Qiagen).

**Figure 1:**
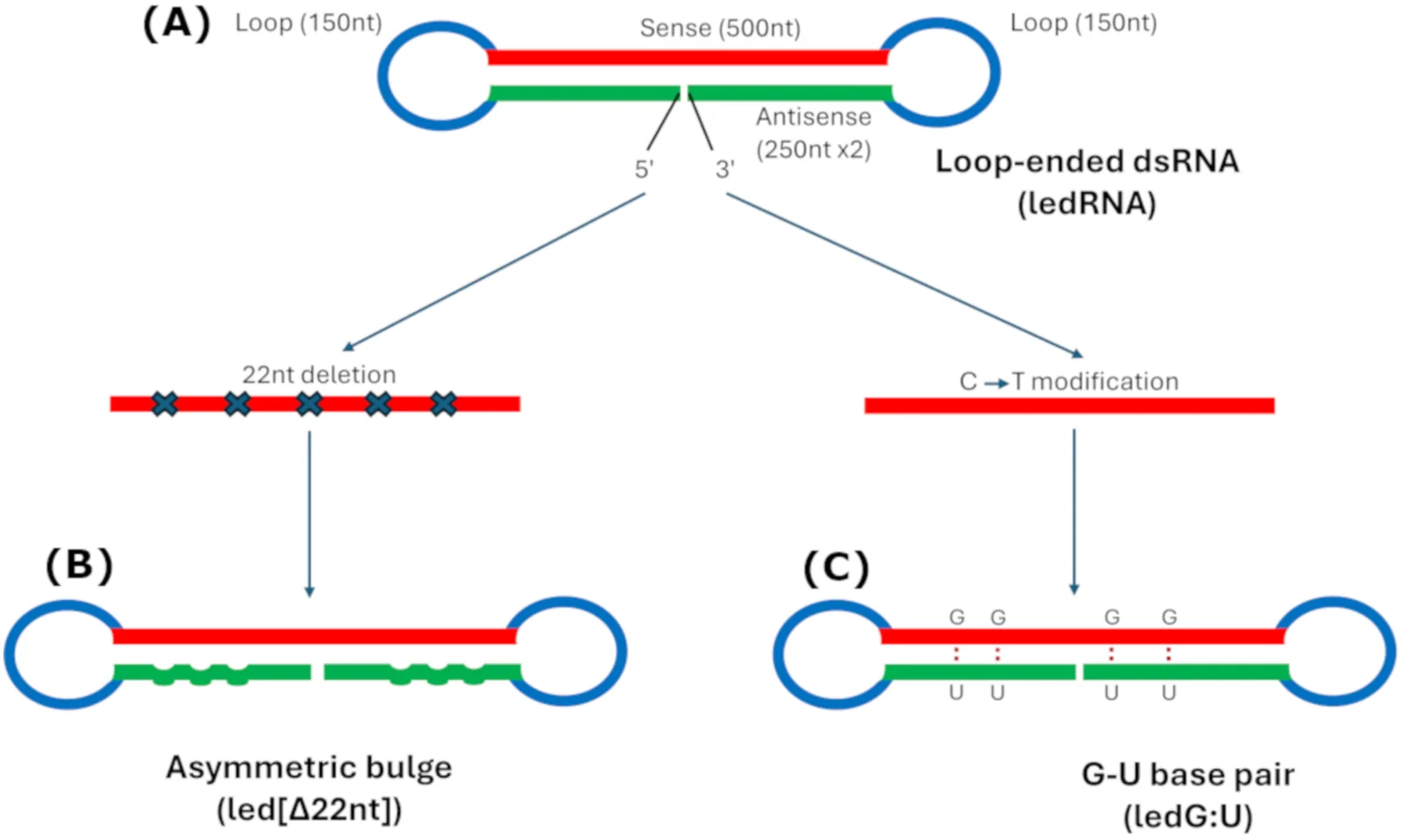
Schematic illustration of the **(A)** ledRNA, **(B)** led[Δ22nt] and **(C)** ledG:U designs. Red lines denote sense strands complementary to the target mRNA, and green lines denote antisense strands. The asymmetric bulge design incorporates single-nucleotide deletions in the sense strand every 22 nt, creating corresponding bulges in the antisense strand. In the ledG:U design, cytosines in the sense strand are substituted with thymines to generate G-U wobble base pairing.

Loop-ended dsRNA designs incorporating either G-U or asymmetric bulge modifications were designed to target the same 500 bp region used for the conventional dsRNA. The ledRNA architecture consisted of a 500 bp stem flanked by two 150 nt single-stranded loops (**Figure 1A**). For the asymmetric bulge design (led[Δ22]), a single nucleotide deletion was introduced every 22 nt in the sense strand, generating a corresponding bulge on the antisense strand without altering its sequence (**Figure 1B**). For the G-U design (ledG:U), all cytosines in the sense strand were substituted with thymines to create G-U wobble base pairs throughout the stem (**Figure 1C**). A ledGFP was also prepared and used as a negative control. All dsRNA sequences are listed in **Supplementary Table 2**.

Plasmid constructs incorporating advanced designs [ledG:U, led(Δ22nt) and ledGFP] were synthesized by Synbio Technologies (Monmouth Junction, NJ, USA). Linearized plasmids and purified PCR amplicons were used as templates for *in vitro* transcription with the Transcript Aid T7 High Yield Transcription Kits (Thermo Fisher Scientific) and purified using MEGAclear™ Transcription Clean-Up Kit (Thermo Fisher) following the manufacturer’s instructions. Two transcription reactions were performed. A standard transcription reaction containing the manufacturer-supplied NTP mix, which was used for mite bioassays. A Cy3-labelled transcription reaction was used exclusively for the dsRNA adult bee-stability assays, in which 1 µL of unlabeled UTP was replaced with 1 µL of Cy3-UTP (Cytiva). The quality and integrity of dsRNA were verified via 1% agarose gel electrophoresis and stored at −80 °C until further use.

### 2.3 dsRNA stability in solution

Groups of ten newly emerged worker bees were collected from brood frames and transferred into ventilated plastic cages (10 cm³). Bees were maintained at 32 °C and 75% relative humidity in the dark. Each cage was provided with 400 µL of 50% (w/v) sucrose solution containing ledGFP, hpGFP, or dsRNA-GFP at a final concentration of 1 µg/µL, with three cages per treatment. Two microliter aliquots of the dosed diet were collected every hour for 10 hours and electrophoresed on 1% agarose gels prepared in 1× TAE buffer at 100 V for 25 minutes. Gels were visualized using Pop-Bio Vü-F Fluorescence System. Band intensity and fragment size were used to assess relative dsRNA abundance and integrity, with quantification performed in ImageJ (version 1.54g) relative to 0-hour samples for each treatment.

### 2.4 dsRNA stability in adult bees

Cages of adult bees were set up as in section 2.3, except that the sucrose solution was supplemented with dsRNA-GFP, ledGFP, ledG:U-GFP, or led[Δ22nt]-GFP tagged with Cy3-UTP. Two bees per treatment were sampled every 24 hours for four days and stored at −80 °C until analysis.

#### 2.4.1 RNA extraction for Cy3 bee samples

Total RNA was extracted from individual adult bees using the Quick-RNA™ Tissue/Insect Micro prep Kit (Zymo Research), following the manufacturer’s protocol with modifications to minimize photodegradation of Cy3-labelled RNA used in fluorescence-based stability assays.

Each bee was placed into a ZR Bashing Bead Lysis Tube (2 mm beads) (Zymo Research) containing the kit lysis buffer and homogenized at 6000 rpm for 2 × 30 s. To prevent loss of Cy3 signal, all steps following homogenization were conducted under minimal light exposure. Tubes were wrapped in aluminum, and manipulations were performed under dim lighting. Homogenized lysates were clarified by centrifugation at 12,000 *g* for 1 min, and the supernatant was transferred to Zymo-Spin™ columns following the manufacturer’s protocol. RNA was eluted in 60 µL of nuclease-free water, which was kept shielded from light immediately after collection. RNA concentration and purity were measured using a NanoDrop™ spectrophotometer, samples were kept covered until immediately before analysis.

#### 2.4.2 Agarose gel electrophoresis and fluorescence detection of Cy3-labelled RNA

RNA integrity and Cy3 fluorescence stability were assessed by electrophoresing RNA samples on unstained 1% agarose gels prepared in 1× TAE buffer. Samples were mixed with 80% glycerol in place of loading buffer and no dyes added to avoid interference with Cy3 fluorescence. Gels ran at 100 V for 15 minutes. Immediately after electrophoresis, gels were scanned using a fluorescence imaging system Typhoon Trio with Cy3-appropriate excitation/emission settings (~550/570 nm). Gels were shielded from light during and after the run and imaged immediately to minimize photobleaching. Band intensity was quantified using ImageJ (version 1.54g) relative to the band with the highest intensity in the day one sample of each treatment.

### 2.5 Mite immersion bioassay

The dsRNA delivery to Varroa mites was performed using a modified immersion protocol described in Campbell, Budge (29). In brief, groups of four adult mites, collected from brood cells, were immersed in 20 µL of dsRNA solution containing 1 µg/µL of conventional dsRNA, ledG:U or led[Δ22] prepared in 0.9% NaCl. Control treatments included mites immersed in 0.9% NaCl alone or ledGFP. The immersion was conducted in 1.5 mL tubes with 5 tubes per treatment, ensuring mites were completely submerged in the solution, and incubated for 16 hours at 4 °C. Mites were then transferred onto moist filter paper and allowed to recover for 3 hours at 30 °C and 75% relative humidity. Live mites were transferred onto white-eye stage pupae with a fine paintbrush, four mites per pupa, within a size 2 cellulose capsules (Wananfu, China) and maintained in the dark at 30 °C and 75% relative humidity. Mite survival was assessed by recording the number of mites attached to pupae or moving within the capsule, and by confirming movement when transferred to filter paper. Mortality was calculated as the percentage of mites dead per treatment. After 48 hours, surviving mites were collected for gene expression analysis and stored at −80 °C.

### 2.6 Oral-delivery bioassay

Groups of 10 newly emerged worker bees were collected from brood frames and placed into ventilated plastic cages (10 cm^3^) and maintained at 32 °C and 75% relative humidity. For this assay, mites were collected from infested colonies using a powdered sugar method mentioned earlier. Collected mites were transferred into petri dishes lined with filter paper and transported to the laboratory. Fifteen live mites were then introduced onto bees for each cage. A single 200 µL treatment of 50% sucrose syrup containing either conventional dsRNA, ledG:U or led[Δ22] was fed to each cage to deliver 1µg dsRNA per bee targeting either CHH or Pero genes. Cages receiving 50% sucrose syrup alone or ledGFP served as controls.

For each treatment, two replicate cages were established, with one cage designated for sampling at 48 hours and the other for sampling at 72 hours. All live mites from the designated cages were collected at either 48 hours or 72 hours and stored at −80 °C for downstream analysis.

### 2.7 RNA extraction of Varroa mites

Total RNA was extracted from individual Varroa mites using TRIzol™ Reagent (Thermo Fisher Scientific) as described in Damayo, McKee (39). In brief, each mite was placed in a 1.5 mL RNase-free microcentrifuge tube containing 100 µL TRIzol and homogenized using a sterile RNase-free plastic pestle. The homogenate was incubated at room temperature for 5 min to ensure complete dissociation of nucleoprotein complexes. Chloroform (20 µL) was added, and the mixture was vigorously vortexed for 15 s and incubated for 3 min at room temperature, followed by centrifugation at 12,000 *g* for 15 min at 4 °C. The aqueous phase was carefully transferred to a new tube, and RNA was precipitated by adding 50 µL isopropanol and 1 µL of glycogen over night at -20 °C. The RNA was pelleted and washed twice with 75% ethanol, air-dried for 5 min, and dissolved in 10 µL RNase-free water. RNA concentration was determined using NanoDrop™ spectrophotometer.

### 2.8 Gene expression analysis of Varroa mites

Gene expression and dsRNA uptake were assessed by qPCR using the SensiFAST™ SYBR^®^ No-ROX One-Step Kit (Bioline) according to the manufacturer’s instructions. Cycling conditions were as follows: reverse transcription at 45 °C for 10 min, initial denaturation at 95 °C for 2 min, followed by 35 cycles of 95 °C for 15 s, 60 °C for 30 s, and 72 °C for 45 s, with a final extension at 72 °C for 5 min. The Varroa succinate dehydrogenase A (SDHA) was used as a reference gene (37). Each biological replicate represents RNA extracted from an individual mite. qPCR reactions were performed in technical duplicates and averaged prior to analysis. Relative expression was determined using the ΔΔCt method, normalized to succinate dehydrogenase (SDHA), and expressed relative to the untreated control. All primer sequences are listed in **Supplementary Table 1**.

### 2.9 Small RNA sequencing and analysis

Total RNA was extracted as described above from surviving mites from the mite immersion bioassay (Section 2.5) and subjected to small RNA sequencing. Libraries were created using QIAseq™ miRNA Library Kit and sequenced on a DNBSEQ-G400 platform (MGI Tech Co., Ltd., Shenzhen, China) at Qiagen.

small RNA sequencing reads were adapter-trimmed using cutadapt, after which reads of 18–30 bp were retained. Filtered reads were aligned to the target gene sequences LOC111244764 (Pero) and LOC111249228 (CHH) using the Bowtie2 alignment method implemented in Geneious Prime^®^ 2021.2.2, with end-to-end alignment and medium sensitivity settings. To facilitate accurate detection of dsRNA-derived siRNAs from both sense and antisense strands, reads were additionally aligned to modified Pero (XM_022792159) and CHH (XM_022802805) reference sequences incorporating the sense strands of the ledG:U and led[Δ22] designs. A custom Python script was used to generate strand-specific coverage profiles and siRNA length-distribution plots.

### 2.10 Statistical analysis

Stability and gene silencing data were assessed using one-way or two-way ANOVA, as appropriate, followed by Tukey’s post-hoc tests for pairwise comparisons with statistical significance defined as *p* < 0.05. All ANOVA analyses were conducted in GraphPad Prism version 10.5.0.

Mortality data were analyzed as binomial outcomes (dead vs alive). Pairwise comparisons between each treatment and the control were performed using two-tailed Fisher’s exact tests. Statistical significance was defined at *p* < 0.05. Analyses were performed in Python (Python Software Foundation, 2023), and results are presented as percentages with Wilson binomial confidence intervals.

## 3 Results

### 3.1 Advanced dsRNA molecules show improved stability in solution and in adult bees

**Figure 2:**
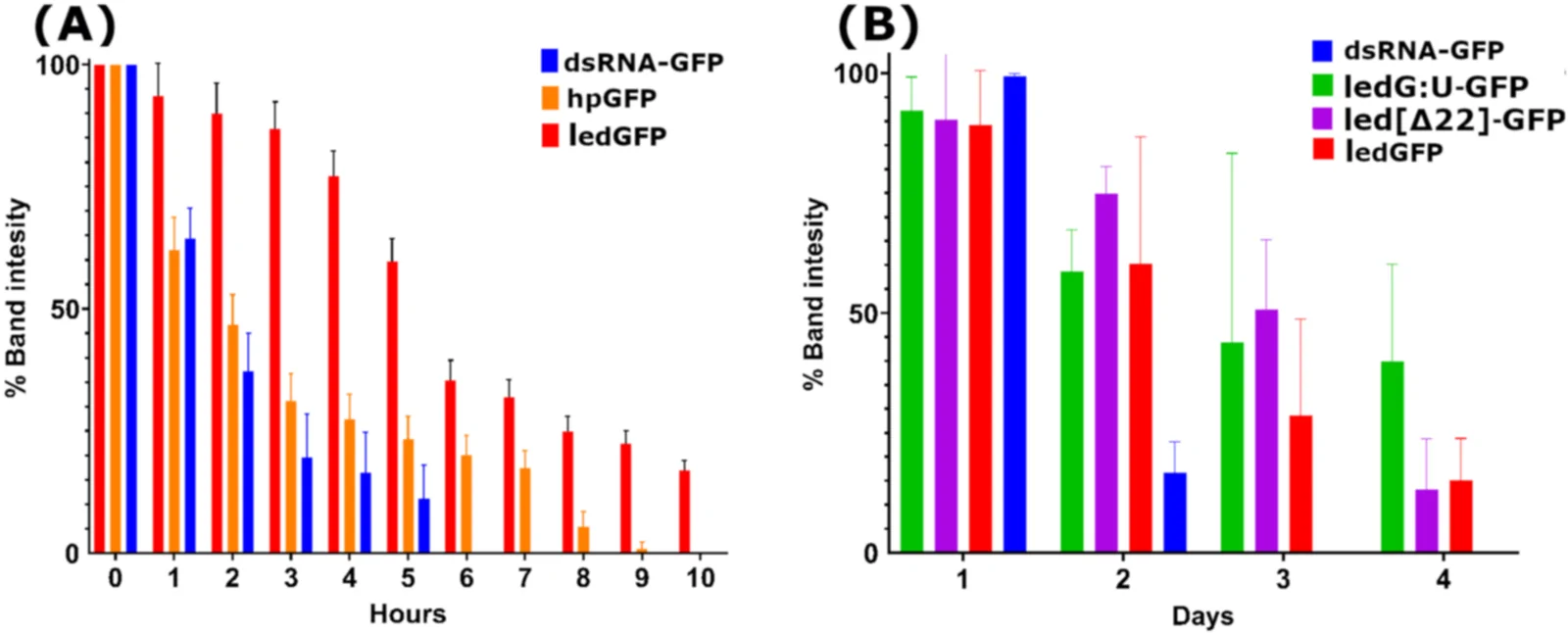
Relative stability of conventional and advanced dsRNA molecules (1 µg/µL) in **(A)** 50% sucrose solution and **(B)** adult worker bees. Statistical analyses for panels A and B were conducted using a two-way ANOVA with Tukey’s multiple comparison test. In panel A, significant pairwise differences were detected for dsRNA-GFP vs. hpGFP (p < 0.001), dsRNA-GFP vs. ledGFP (p < 0.001), and hpGFP vs. ledGFP (p < 0.001). In panel B, significant differences were observed for dsRNA-GFP vs. ledGFP (p = 0.034), dsRNA-GFP vs. ledG:U-GFP (p < 0.001), and dsRNA-GFP vs. led[Δ22]-GFP (p = 0.0004). Data are presented as mean relative signal (percent relative to initial intensity) ± SD quantified using ImageJ.

To determine whether the advanced molecules improve dsRNA stability, persistence was measured in 50% sugar solution and in adult bees over time using Agarose gel electrophoresis of labelled RNA (**Figure 2**). In sugar solution, the conventional dsRNA degraded rapidly with the band intensity decreasing by ~35% within the first hour, ~75% by three hours and becoming undetectable by 6 hours. Hairpin RNA (hpRNA) showed a moderate improvement retaining ~25% band intensity at 6 hours and remaining detectable until the 9-hour mark. In contrast the loop-ended RNA (ledRNA) degradation was substantially slower, retaining ~25% intensity at 9 hours and ~18% at 10 hours. Overall, ledRNA remained approximately three-fold more abundant than conventional dsRNA between 4 and 6 hours (**Figure 2A**).

A similar trend was observed following delivery into adult bees. Conventional dsRNA declined rapidly, falling to ~18% of the initial signal by day 2 and becoming undetectable by day 4. In contrast, ledRNA decreased to ~60% of its initial intensity by day 2, ~30% by day 3, and ~15% by day 4. The led[Δ22] construct showed a similar pattern, retaining 70%, 50%, and 12% of its initial signal on days 2, 3, and 4, respectively. The G-U modified dsRNA exhibited improved persistence, retaining approximately 58%, 44%, and 40% of its initial signal on days 2, 3, and 4, respectively. Overall, the advanced constructs exhibited significantly greater persistence than conventional dsRNA (dsRNA-GFP vs. ledGFP (p = 0.034), dsRNA-GFP vs. ledG:U-GFP (p < 0.001), and dsRNA-GFP vs. led[Δ22]-GFP (p = 0.0004).(**Figure 2B**).

### 3.2 Immersion in advanced dsRNA molecules produced higher Varroa mite mortality and gene silencing

**Figure 3:**
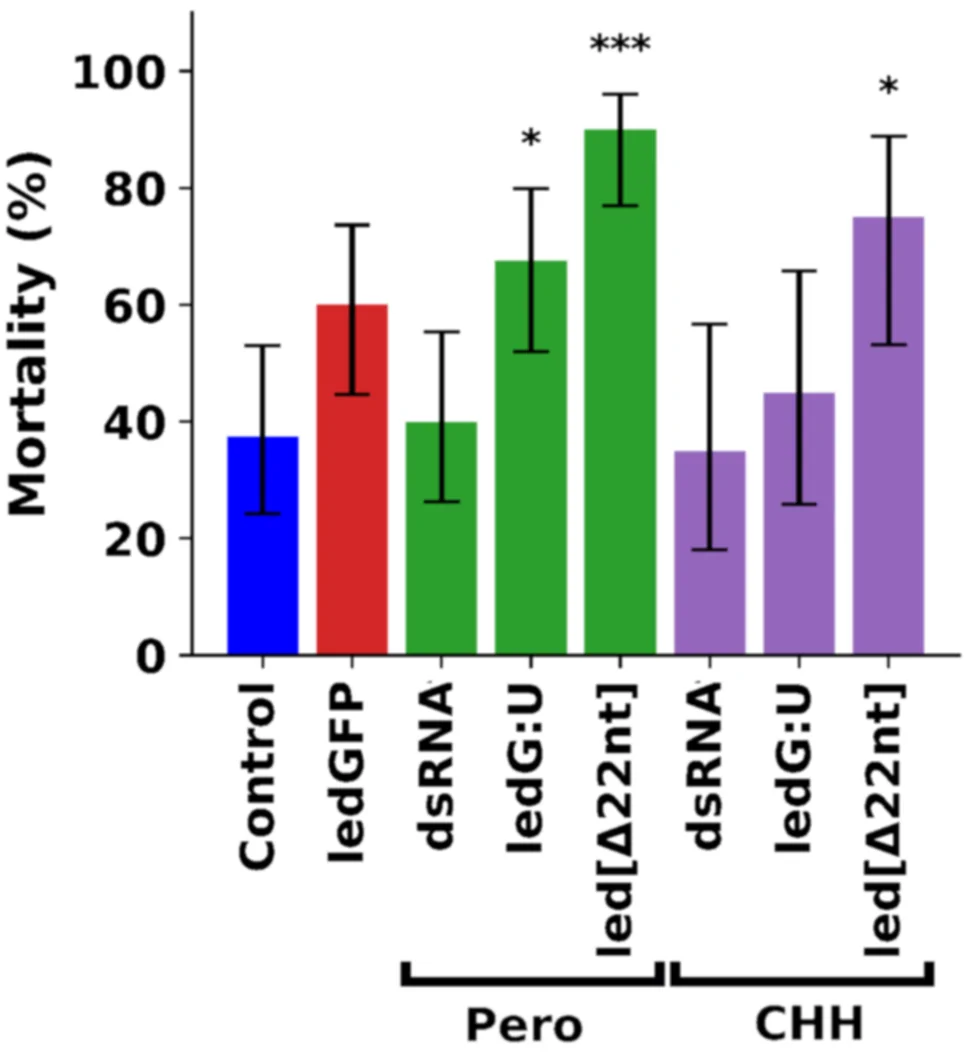
Varroa mite mortality at 48 hours following immersion 0.9% NaCl (Control), 1 µg/µl of ledGFP or the three dsRNA designs solution targeting either Pero or CHH genes. Mortality was calculated as the proportion of dead mites per treatment. Error bars indicate 95% Wilson binomial confidence intervals. Statistical comparisons between each treatment and the control were performed using two-tailed Fisher’s exact tests. Significance is indicated as p < 0.05.

The efficacy of advanced dsRNA molecules targeting two Varroa genes, Pero and CHH, was first tested by immersing mites directly in dsRNA solution. These bioassays showed that mite mortality at 48 hours was higher for both gene targets with the advanced dsRNA molecules compared with conventional dsRNA (**Figure 3**). Relative to the untreated control (37.5% mortality), led[Δ22]-Pero and led[Δ22]-CHH significantly increased mortality by 52.5% (p < 0.001) and 37.5% (p = 0.0127), respectively, followed by ledG:U-Pero, which increased mortality by 30%(p = 0.0133). In contrast, conventional dsRNA showed no elevation in 48-hour mortality above the untreated control for both genes. The ledGFP treatment also produced an unexpected 22.5% (p = 0.0729) increased mortality, indicating a non-specific response to this design. No statistical data is available for this experiment due to a single replicate structure for each treatment.

**Figure 4:**
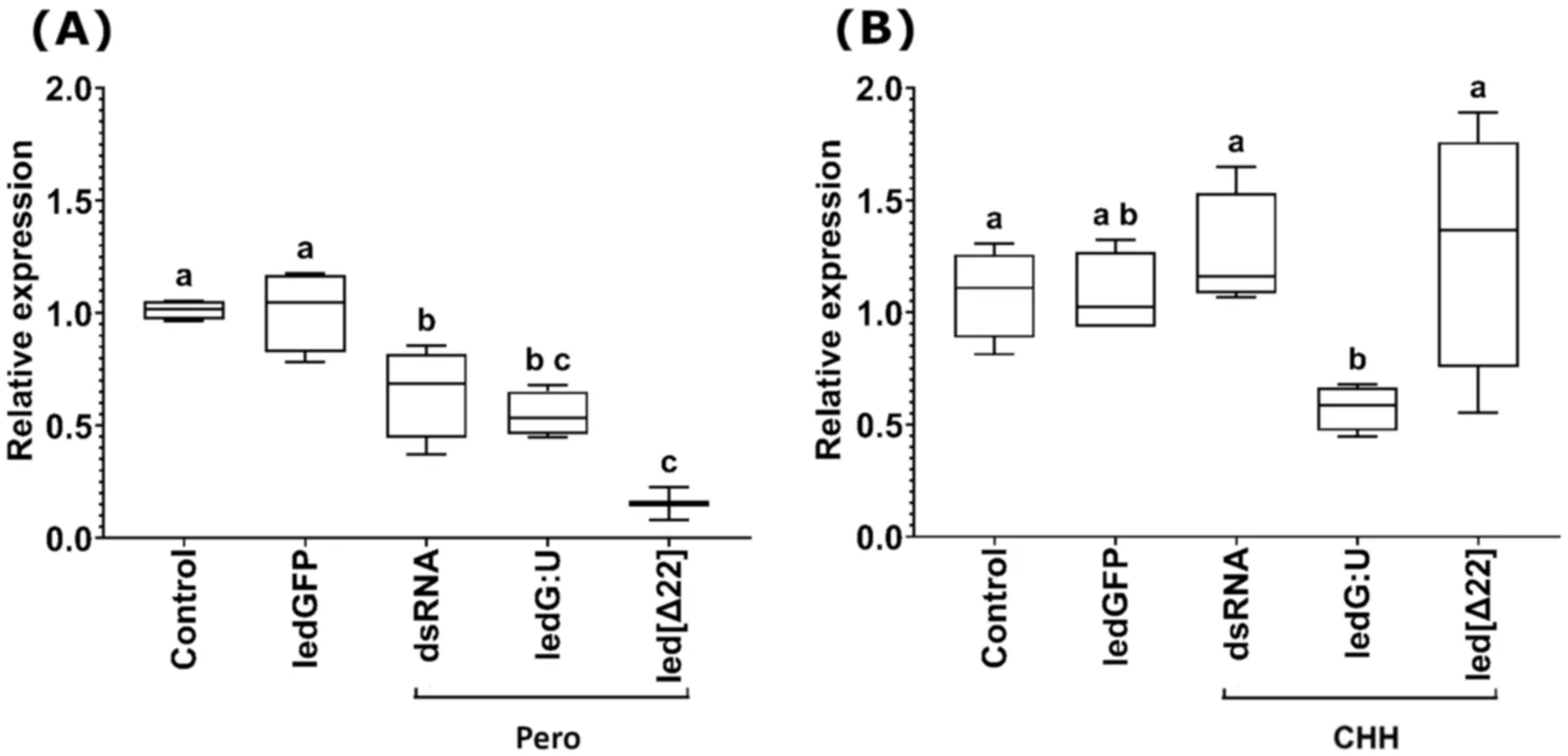
Relative gene expression of **(A)** Pero, **(B)** and CHH genes in Varroa mites 48 hours after immersion in dsRNA solutions. dsRNA treatments included conventional dsRNA, ledG:U, and led[Δ22] molecules, with a ledGFP control. Data represent mean ± SEM for n = 5 biological replicates except led[Δ22]-Pero and led[Δ22]-CHH which had 2 and 4 biological replicates, respectively. Significant differences between treatments as determined by Tukey’s HSD multiple comparison test (p < 0.05) are indicated with different letters.

Varroa mites collected from the immersion bioassays were tested by qPCR to determine the relative expression of the target genes following dsRNA treatments. For Pero, significant gene silencing was observed following treatment with the conventional dsRNA (~36% reduction p = 0.046), the ledG:U (~46% reduction p = 0.036) and most strongly with the led[Δ22nt] (~85% reduction p<0.001) (**Figure 4A**). In contrast, significant gene silencing for CHH was only detected with ledG:U (~47% reduction p = 0.035), despite the observed effects of led[Δ22nt] on mite mortality (**Figure 4B**). Expression of Pero and CHH was unaffected in the untreated and ledGFP controls.

### 3.3 Oral-delivery of advanced dsRNA molecules produced higher Varroa mite mortality and gene silencing

To simulate natural exposure pathways, dsRNA treatments were fed to adult worker bees being parasitized by feeding Varroa mites. At 48 hours, mite mortality above control levels was observed only for the ledG:U-Pero and ledG:U-CHH treatments, each resulting in an 18% (p = 0.158) increase relative to the untreated control, which exhibited a baseline mortality of 7% (**Supplementary Figure 1**). This contrasts with the immersion assay, in which the led[Δ22] designs produced the highest mortality.

**Figure 5:**
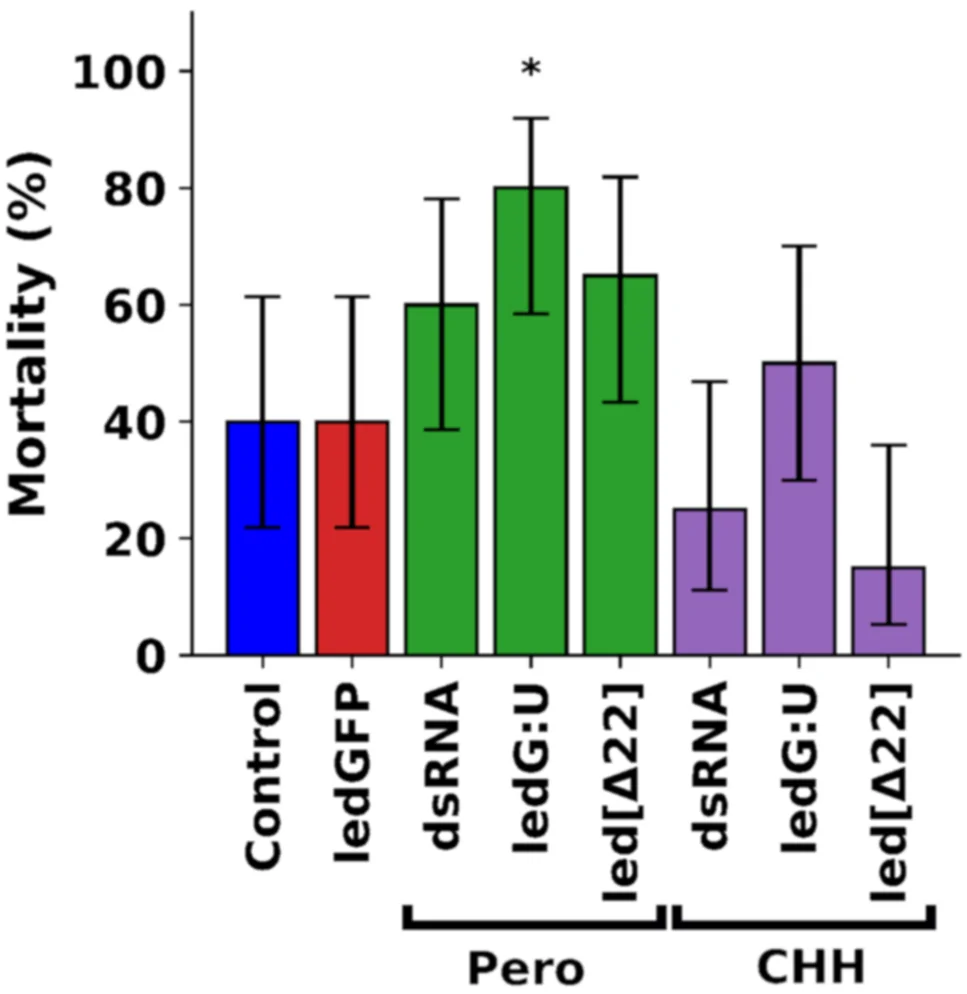
Mite mortality following indirect exposure to dsRNA at 72 hours. Parasitized adult bees were fed 50% sucrose solution dosed with 10 µg of ledGFP or the three dsRNA designs: conventional dsRNA, ledG, U, and led[Δ22] with an untreated control. Mortality was calculated as the proportion of dead mites per treatment. Error bars indicate 95% Wilson binomial confidence intervals. Statistical comparisons between each treatment and the control were performed using two-tailed Fisher’s exact tests. Significance is indicated as P < 0.05.

At 72 hours, mite mortality was elevated for the Pero-targeting designs, with conventional dsRNA, ledG:U, and led[Δ22] associated with 20% (p = 0.346), 40% (p = 0.022), and 25% (p = 0.204) higher mortality, respectively, compared with the untreated control (**Figure 5**). In comparison, conventional dsRNA treatments and advanced designs targeting CHH had little effect on mite mortality, with the exception of ledG:U, which resulted in 15% (p = 0.75) increased mortality.

**Figure 6:**
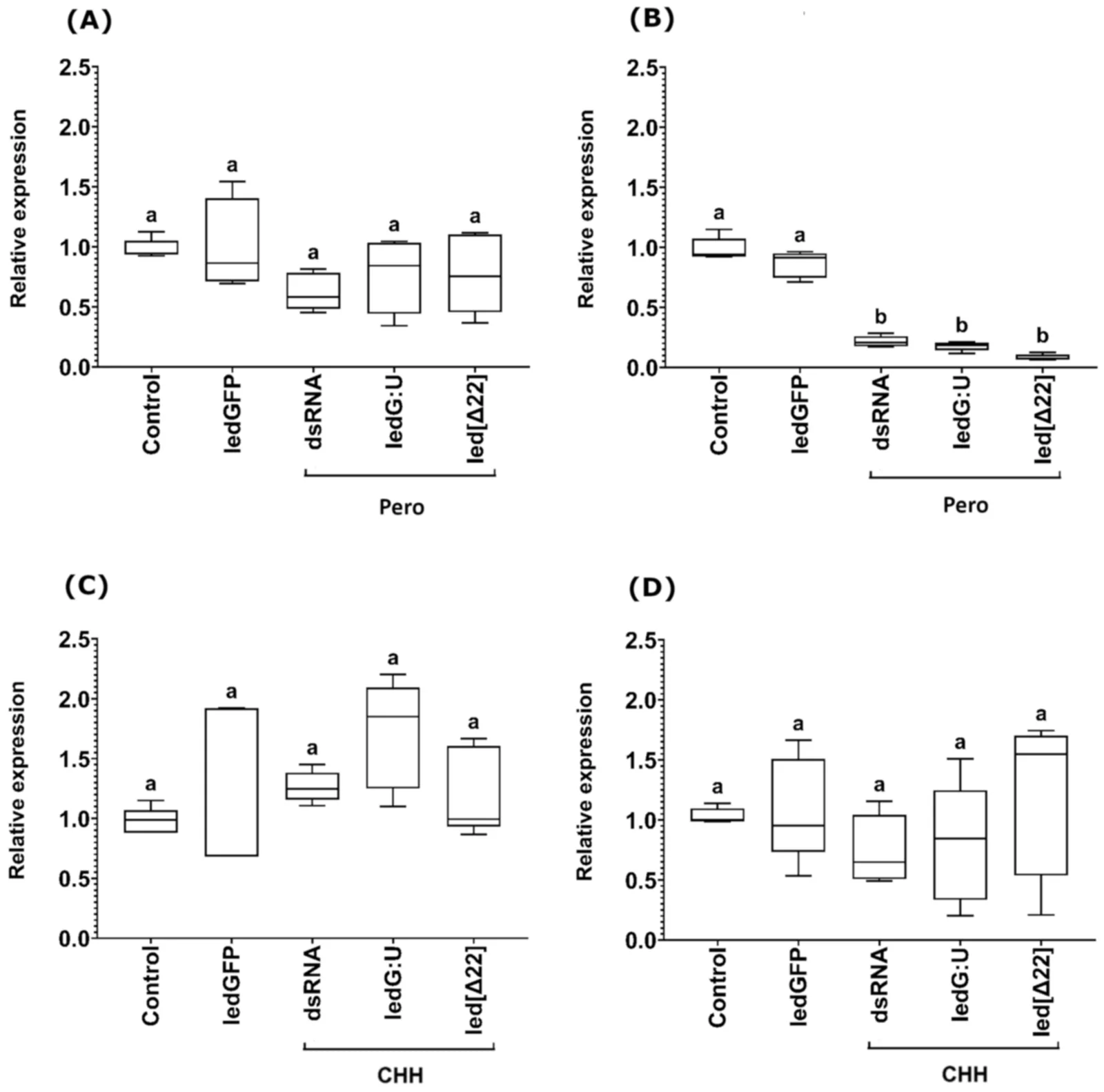
Relative gene expression of Pero **(A, B)** and CHH **(C, D)** genes in Varroa mites at 48 hours **(A, C)** and 72 hours **(B, D)** after bee-mediated delivery of dsRNA treatments. dsRNA treatments included conventional dsRNA, ledG:U, and led[Δ22], with a ledGFP control. Relative expression was determined using the ΔΔCt method, normalized to succinate dehydrogenase (SDHA), and expressed relative to the untreated control. Data represent mean ± SEM for n = 5 individual mites from each treatment. Significant differences between treatments as determined by Tukey’s HSD multiple comparison test (p < 0.05) are indicated with different letters.

Relative gene expression for the two target genes was consistent with the Varroa mite mortality data. Significant gene silencing was only evident for Pero at 72 hours, with all dsRNA designs having a strong silencing effect: 78% for conventional dsRNA (p<0.001), 82% for ledG:U (p<0.001) and 92% for led[Δ22] (p<0.001) (**Figure 6B**). No significant gene silencing was detected for the dsRNA designs targeting CHH at the same 72-hour timepoint (**Figure 6D**). No measurable effect on gene expression was observed at 48 hours for Pero or CHH (**Figures 6A, C**).

### 3.4 LED-G:U and LED[Δ22] constructs generate target-specific siRNAs with defined size distributions and antisense bias

To determine whether differences in biological efficacy were associated with design-dependent processing into functional siRNAs, we analyzed siRNA populations generated in mites immersed in treatment. Sequencing reads (18–30 nt) were mapped to either Pero (LOC111244764) or CHH (LOC111249228).

**Figure 7:**
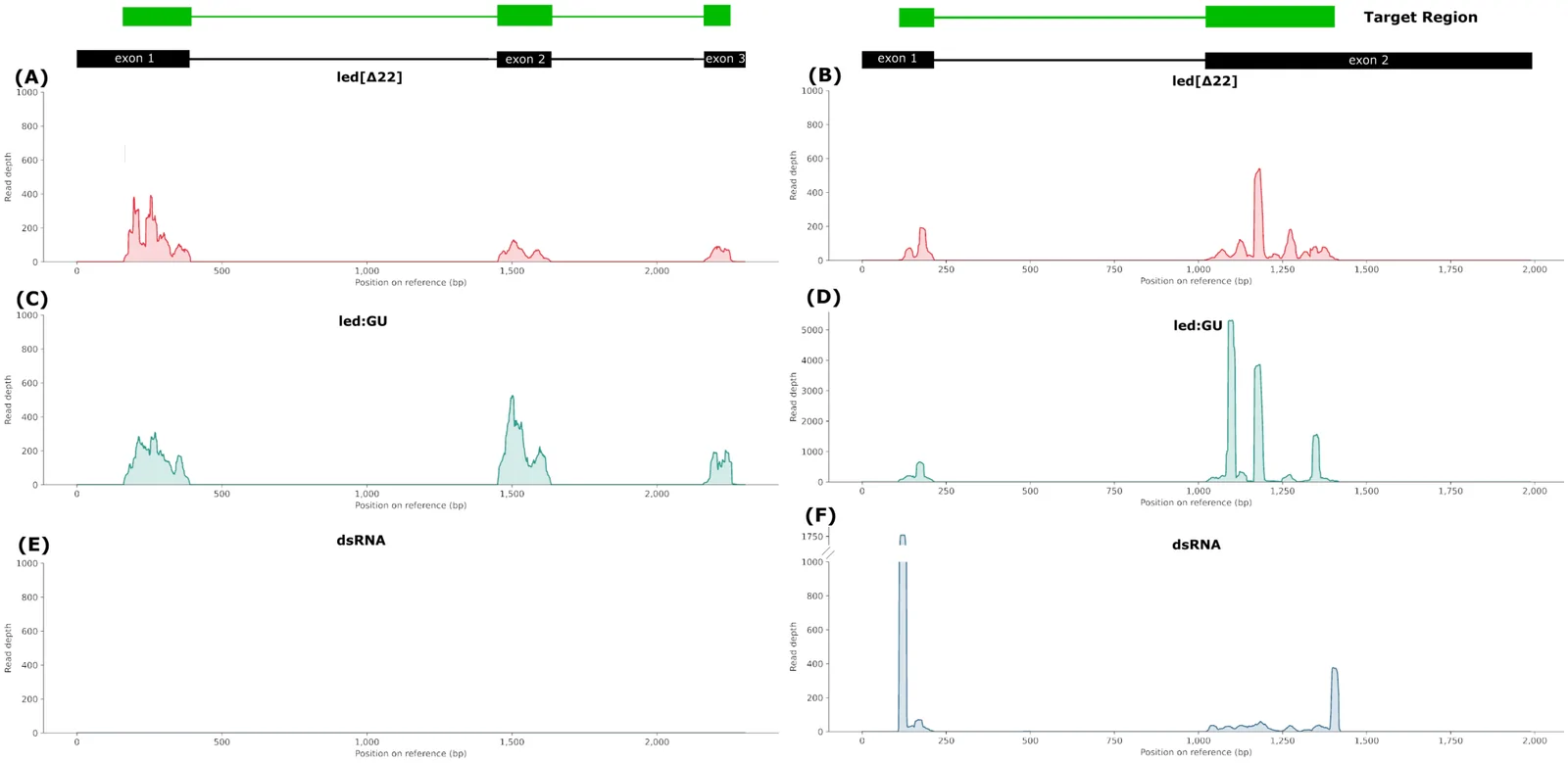
siRNA read (18 - 30 nt) coverage profiles across **(A)** Pero (LOC111244764) and **(B)** CHH (LOC111249228). Mean read depth represents the average across three biological replicates (n = 3). Coverage profiles are shown for each dsRNA design, including led[Δ22], ledG:U, and dsRNA.

For the Pero gene, the led[Δ22] design showed moderate coverage within the target region, with mean read depth reaching a maximum of 415 (**Figure 7A**). In contrast, the ledG:U treatment produced higher-depth coverage, with mean read depth peaking at 565 (**Figure 7C**). Conventional dsRNA coverage was very low a read depth of for Pero (**Figure 7E**). Across all designs, coverage spanned 100% of the target region sequence, consistent with siRNA production being confined to the region targeted by the dsRNA design rather than distributed across the full locus.

For the CHH gene, a similar but more pronounced pattern was observed. The led[Δ22] design again showed relatively low-depth coverage within the target region, with mean read depth reaching a maximum of 587 (**Figure 7B**). In contrast, the ledG:U treatment generated substantially higher-depth coverage that was concentrated across the target region, with mean read depth peaking at 5,412 (**Figure 7D**). Unlike Pero, conventional dsRNA targeting CHH produced detectable coverage, with a maximum mean read depth of 1,769; however, this signal was largely restricted to a narrow region of the target sequence rather than being evenly distributed (**Figure 7F**). Across all designs, coverage spanned 100% of the CHH target, again indicating that siRNA production was localized to the targeted region.

**Figure 8:**
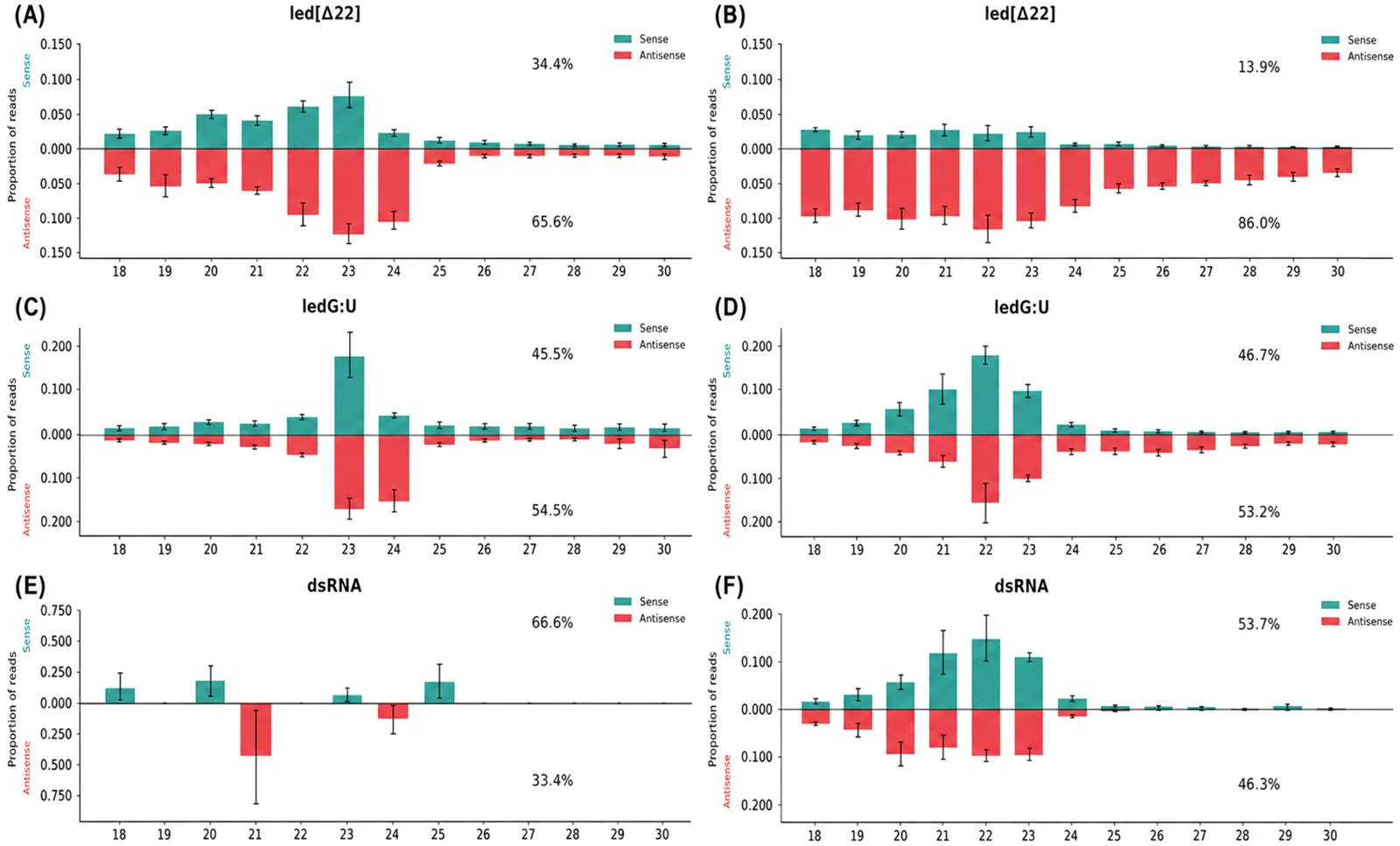
Read-length distributions (18-30 nt) of siRNAs mapping to modified Pero XM_022792159 **(A, C, E)** and CHH XM_022802805 **(B, D, F)** reference sequences for each dsRNA design [dsRNA, ledG:U, and led(Δ22)]. For each replicate, read counts for each size class and strand were normalized to the total number of mapped reads in that replicate (sense and antisense combined). Bar heights represent the mean proportion of reads across replicates.

Sorting target-mapped siRNAs by read length revealed a strong predominance of antisense reads across most size classes for both advanced dsRNA designs, consistent with the generation of siRNAs from the introduced dsRNA designs (**Figure 8**). For the led[Δ22] design, antisense reads accounted for 65% and 85% of all mapped reads for Pero and CHH, respectively (**Figures 8A, B**), whereas antisense reads comprised 54.5% and 53.2% of mapped reads for ledG:U targeting Pero and CHH (**Figures 8C, D**). In contrast, conventional dsRNA read distributions were skewed toward the sense strand, with 46.7% sense reads and 46.3% antisense reads, indicating a reduced antisense bias relative to the advanced designs (**Figure 8F**).

Analysis of siRNA length distributions across the target region further highlighted design and target dependent differences. For Pero, siRNAs derived from advanced dsRNA designs showed a dominant peak at the 23 and 24 nt size class (**Figures 8A, C, E**), whereas CHH-derived siRNAs exhibited a shift toward 22 and 23 nt peaks (**Figures 8D, F**). In most cases, the predominant antisense size classes were accompanied by corresponding sense peaks, consistent with Dicer processing of dsRNA into sense-antisense siRNA duplexes. However, the led[Δ22]-CHH (**Figure 8B**) design had a broader size class distribution without a sense strand peak, suggesting less effective processing and more degradation. Overall, these data demonstrate design-dependent differences in siRNA production and strand distribution, with the asymmetric bulge and G-U modified designs show robust and consistent processing into functional siRNAs.

## 4 Discussion

RNAi-based biopesticides are emerging as a viable strategy in pest management but their broader adoption requires that dsRNA can effectively avoid degradation in the external and internal environments to reach their gene targets. In this study, we used the honey bee-Varroa pathosystem to demonstrate that three novel dsRNA modifications can exhibit enhanced stability, stronger efficacy, and greater processing into siRNA populations compared with conventional dsRNA. The loop-ended structure stabilised the dsRNA when fed to adult bees, remaining intact for twice as long in adult bees. When loop-ended structures were coupled with G-U and asymmetric bulge dsRNA sequence modifications, processing into siRNAs and biological efficacy were higher when targeting two Varroa genes. Together, these findings highlight that advanced dsRNA designs can address key challenges for the field application of RNAi and provide the first demonstration of these molecules effectively targeting Varroa mites.

The ledRNA design substantially improved dsRNA stability in both the sucrose feeding media and within adult bees. While dsRNA is generally stable when stored in solution, it degrades quickly when exposed to bees under hive conditions (23). Loop-ended designs have emerged as a key strategy for reducing nuclease-mediated degradation by protecting the dsRNA ends from exonuclease activity while preserving the double stranded stem required for Dicer processing (18, 40). Our results mirror those of Ge et al. (40), who reported that ledRNA outperformed hpRNA and linear dsRNA when fed orally to small brown planthoppers. The enhanced stability of ledRNA could enable more intact molecules to persist in the insect gut and haemolymph. Improved systemic activity of ledRNA over hpRNA and conventional dsRNA is demonstrated in plants (18) and may also apply to insects and arachnids. These attributes are particularly valuable in the context of Varroa, as an obligate parasite feeding on honey bees. Linear dsRNA is shown to be effectively transferred to Varroa when fed to bees (23); therefore, the greater uptake and stability of ledRNA within the bee host increases the likelihood that feeding mites will acquire sufficient intact dsRNA to trigger RNAi-mediated gene silencing. Using the ledRNA architecture offers a promising structural foundation for developing improved oral dsRNA interventions against Varroa in managed bee hives.

The target gene Pero had the best overall efficacy with both the asymmetric and G-U designs producing greater gene silencing (46-92%) and higher mite mortality (up to 52%) compared with conventional linear dsRNA after 48 hours. A recent study targeting Pero with conventional dsRNA also reported strong gene silencing but had slower effects on mite mortality, only reaching 40-50% mortality after five days (30). Our results for CHH were more variable with only the G-U design inducing both gene silencing (47%) and mite mortality (10-40%). This efficacy was comparable with a previous study where conventional dsRNA resulted in ~55% gene silencing at 48 hours and ~30% mortality observed after 96 hours (37). Notably, these effects were achieved using a higher 2.5 µg/µL dsRNA concentration compared with the 1 µg/µL concentration applied in our study. We also note that mite mortality in our untreated and ledGFP controls was higher across assays than these previous studies. The immersion assay method was relatively stressful on mites, involving overnight immersion in solution at 4 °C, although this was successfully used by Campbell, Budge (37). A shorter immersion time at room temperature, as used by Becchimanzi, Cacace (30) may have helped reduce treatment stress on mites. Testing with ledRNA control genes in addition to GFP would also help examine any non-target effects from the ledRNA design on mortality. Overall, our results highlight the advanced dsRNA designs can provide improved RNAi efficacy, potentially at reduced concentrations, although further optimisation of gene-design combinations is still needed.

To explore design-dependent differences in RNAi processing in Varroa, we examined the siRNA populations generated from each construct. Past studies on Varroa’s antiviral response have shown that dsRNA is processed into defined 21–24 nt siRNA populations, with a clear 24 nt antisense siRNA bias (39, 41). In this study, the Pero-targeting G-U and asymmetric bulge designs generated this signature profile with peaks of antisense-biased 23–24 nt siRNAs across the target gene region, reflective of effective RNAi processing in Varroa. In contrast, CHH-targeting designs produced a shifted siRNA distribution that peaked at 22–23 nt. A similar profile was observed for the ledGFP control (**Supplementary Figure 2**), suggesting there may only have been a primary Dicer-produced siRNA response to these constructs. In nematodes, secondary siRNA amplification is mediated by RNA-dependent RNA polymerase (RdRP), which generate distinct size classes and expand coverage beyond the initial target region by using mRNA as a template (42). While secondary siRNAs were not detected downstream of the target region in this study, the presence of 24 nt antisense-biased siRNA populations from the Pero-targeting designs could be evidence of RdRP-associated amplification. It may also suggest that RNAi efficacy could be driven by target-dependent differences in dsRNA processing into siRNAs and engagement of RdRP. Several RdRP homologs have been identified in Varroa (43), although their functional role in secondary siRNA amplification following exogenous dsRNA requires further investigation.

In our assays, the G-U design showed comparatively consistent performance, coinciding with robust target-specific siRNA production and an antisense bias. The G-U strategy has been used in plants to mitigate transgene self-silencing caused by inverted-repeat architectures (19), but other mechanisms likely apply to exogenously delivered dsRNA in insects and mites. One possibility is that G-U wobble pairing introduces an imperfect duplex that alters substrate recognition and cleavage kinetics by arthropod Dicer-2. Dicer processing is sensitive to duplex structural features, including mismatches and termini composition (44, 45). Internal mismatches such as G-U pairing introduce structural distortions that can modulate Dicer binding thus reducing rapid processing to change the balance between cleavage and persistence of intact dsRNA. In the honey bee–mite system, increased persistence could have multiple benefits, including improved stability within feeding solutions and adult bees and potentially longer availability to feeding mites.

The asymmetric bulge design has been used to increase RNAi efficacy in transgenic plants by shifting siRNA size-class profiles (22). In these systems, a 22-nt bulge configuration can increase the abundance of 22-nt siRNAs, which are associated with secondary siRNA amplification (22). Here, we tested a 22-nt bulge configuration based on its superior performance in plants; however, in our system this design did not produce a discrete 22-nt siRNA peak for either gene target. This divergence likely reflects fundamental differences in dsRNA processing between plants and arthropods. Plants encode multiple specialized Dicer-like enzymes (e.g., DCL2 and DCL4) that generate functionally distinct 21-nt and 22-nt siRNAs (46), whereas arthropods predominantly rely on Dicer-2, which processes dsRNA into a narrower size range without pronounced size-specific functional diversification (44). Optimizing bulge spacing to better match the dominant Varroa size classes (e.g., 23-nt or 24-nt) may better align with endogenous processing to increase the 24-nt component, consistent with the 24-nt antisense bias reported for Varroa antiviral responses (39, 41). The improved efficacy of the asymmetric bulge design observed here may mostly reflect the enhanced dsRNA stability enabling sustained siRNA production, rather than any influence on siRNA size classes. Further testing of alternative bulge configurations will be needed to resolve these possibilities.

Taken together, our findings from this study suggest the advanced designs could improve RNAi outcomes against Varroa however, confirmation in colony and field level settings will be necessary to verify their practical utility.

## Data Availability

The original contributions presented in the study are publicly available. This data can be found here: CSIRO Data Collection: https://data.csiro.au/collection/csiro:76119.
