# Supplementary material for: Novel dsRNA designs increase the stability and bioefficacy of RNAi-based pesticides for Varroa destructor
Source: Front Insect Sci. 2026 Aug 17;6:1868457. doi: 10.3389/finsc.2026.1868457 (PMC13527466; doi:10.3389/finsc.2026.1868457)
Supplement: Supplementary file 1 [file SupplementaryFile1.docx]

Supplementary data

Supplementary Table S 1: Primer

| Gene/target | Assay Type | Primer Name | Sequence (5`-3`) | Amplicon (bp) | Notes |
| --- | --- | --- | --- | --- | --- |
| Peritrophin-A (Pero) | qPCR | Pero-F | TCTCATCCATGTCAGCAGCA | 100 | Gene-specific |
|  |  | Pero-R | CACAATTGAAGGCAGCGCAA |  |  |
|  |  | T7-Pero-F | TAATACGACTCACTATAGGGCTGTAAAGGTGAATTTGTC | 500 | T7 promoter added |
|  |  | T7-Pero-R | TAATACGACTCACTATAGTTAATAAAACCACTTCTTCGCAGAA |  |  |
| crustacean hyperglycemic hormone (CHH) |  | CHH-F | TGGGTTGAAGCAAAGGCAAC | 133 | Gene-specific |
|  |  | CHH-R | GGATTGGTAAACGACGCTCTC |  |  |
|  |  | T7-CHH-F | TAATACGACTCACTATAGGGCTGTAAAGGTGAATTTGTC | 500 | T7 promoter added |
|  |  | T7-CHH-R | TAATACGACTCACTATAGTTAATAAAACCACTTCTTCGCAGAA |  |  |
| SDHA (reference) |  | SDHA-F | AAACCGGGAACGACCTTATC | 120 | Housekeeping gene |
|  |  | SDHA-R | TCCAATCCTTCCAACTGTCC |  |  |

Supplementary Table S 2: dsRNA sequences

| Gene/Target | Design | Sequence (5`-3`) | Size | Notes |
| --- | --- | --- | --- | --- |
| Peritrophin-A (Pero) | G-U | CCGTTCGTGCAGCGCCGTGATTCGCTTCTTGCGATGCACAGGAGGCAAATTACTGCGAGATGCATCAAAAACATGTTTCTTGTCATTTTTGAAGTACAAGTGACCGACAAATCGCAAAGAAGGTTATGAGAAACTCCTTCGTTGTGAGTCGAGTGGACTGCAGATATCTGGAGAGCCTAAATAAGCTGTTCAGAAGTTTTCGACGAGTGTTAATATAGCACAATTGAAGGCAGCGCAAAGATGTCCTTACAAUGGACCAGUAAAGAGCAGUCACAUAUCUAUUCCAUAGACUUGCUUCUUUGAAAUUUGGAUACACAGCUCCAAUCCAUGUCUUUCUUGGAUUAGGAUAUCUAUCUGCAAUCAGGUAGUUGAAACAUCCAGCACAAUGUAUAGCGAACAAGTAAGGATATTTTTGTGTTGTTTTTAATTGTGTTATATTAATATTTGTTGAAAATTTTTGAATAGTTTATTTAGGTTTTTTAGATATTTGTAGTTTATTTGATTTATAATGAAGGAGTTTTTTATAATTTTTTTTGTGATTTGTTGGTTATTTGTATTTTAAAAATGATAAGAAATATGTTTTTGATGTATTTTGTAGTAATTTGTTTTTTGTGTATTGTAAGAAGTGAATTATGGTGTTGTATGAATGGATGAATAGTGTTATGTTAAGAATATAATATATTATATAAGTAGATGTATTTTGATGTTAATGATGAAAAGTGTTAGGGTTTTTGGATATGTTATAATGGGATAGTTGTATTATGATGTTGTGGTGTGGGAATGAGATATAGTTTTGAATTTTAGGTTTGTGTGTATGTTAAAGATGTGTATTGTTTAAAATTTGATTATGTTTTTTTGTATATATAATTAAGATTAAATAGATTTAAAGAATGAAAGTAAAAUGGACCAGUAAAGAGCAGUCACAUAUCUAUUCCAUAGACUUGCUUCUUUGAAAUUUGGAUACACAGCUCCAAUCCAUGUCUUUCUUGGAUUAGGAUAUCUAUCUGCAAUCAGGUAGUUGAAACAUCCAGCACAAUGUAUAGCGAACAATTACTTTCGTTCTTTGGATCTATTTAATCTTGGTTGTATATGCAAAGAAGCATGGTCAGATTTTGGGCAGTGCACGTCTTTGGCGTGCACGCAGACCTGGAGTTCGGAACTGTATCTCGTTCCCACACCACAGCATCGTAGTACGGCTGTCCCGTTATAACATGTCCAAAAACCCTGGCACTTTTCGTCGTTGACATCGGGATACATCTGCTTGTGTAGTGTATTATGTTCTTGGCATGACGCTGTTCGT | 1300 |  |
|  | Asymmetric | CCGTTCGTGCAGCGCCGTGATTCGCTTCTTGCGATGCACAGGAGGCAAATTACTGCGAGATGCATCAAAAACATGTTTCTTGTCATTTTTGAAGTACAAGTGACCGACAAATCGCAAAGAAGGTTATGAGAAACTCCTTCGTTGTGAGTCGAGTGGACTGCAGATATCTGGAGAGCCTAAATAAGCTGTTCAGAAGTTTTCGACGAGTGTTAATATAGCACAATTGAAGGCAGCGCAAAGATGTCCTTACAAUGGACCAGUAAAGAGCAGUCACAUAUCUAUUCCAUAGACUUGCUUCUUUGAAAUUUGGAUACACAGCUCCAAUCCAUGUCUUUCUUGGAUUAGGAUAUCUAUCUGCAAUCAGGUAGUUGAAACAUCCAGCACAAUGUAUAGCGAACAAGTAAGGACATCTTTGCGCTGCCTTCAATTGTGCTATATTAACACTCGTCGAAAACTTCTGAACAGCTTATTTAGGCTCTCCAGATATCTGCAGTCCACTCGACTCACAACGAAGGAGTTTCTCATAACCTTCTTTGCGATTTGTCGGTCACTTGTACTTCAAAAATGACAAGAAACATGTTTTTGATGCATCTCGCAGTAATTTGCCTCCTGTGCATCGCAAGAAGCGAATCACGGCGCTGCACGAACGGACGAACAGCGTCATGCCAAGAACATAATACACTACACAAGCAGATGTATCCCGATGTCAACGACGAAAAGTGCCAGGGTTTTTGGACATGTTATAACGGGACAGCCGTACTACGATGCTGTGGTGTGGGAACGAGATACAGTTCCGAACTCCAGGTCTGCGTGCACGCCAAAGACGTGCACTGCCCAAAATCTGACCATGCTTCTTTGCATATACAACCAAGATTAAAAUGGACCAGUAAAGAGCAGUCACAUAUCUAUUCCAUAGACUUGCUUCUUUGAAAUUUGGAUACACAGCUCCAAUCCAUGUCUUUCUUGGAUUAGGAUAUCUAUCUGCAAUCAGGUAGUUGAAACAUCCAGCACAAUGUAUAGCGAACAATTACTTTCGTTCTTTGGATCTATTTAATCTTGGTTGTATATGCAAAGAAGCATGGTCAGATTTTGGGCAGTGCACGTCTTTGGCGTGCACGCAGACCTGGAGTTCGGAACTGTATCTCGTTCCCACACCACAGCATCGTAGTACGGCTGTCCCGTTATAACATGTCCAAAAACCCTGGCACTTTTCGTCGTTGACATCGGGATACATCTGCTTGTGTAGTGTATTATGTTCTTGGCATGACGCTGTTCGT | 1277 |  |
| crustacean hyperglycemic hormone (CHH) | G-U | CGGTTGTGACGCAGTTGGTCTCCTGCTGATTTAAGGGATAGGTCCGTAGAGTTGATTTACCATACTTTCAAGCATATCCTTTTCCTTGTGGAGCATTAGGGCTTCAGTGCACAACAAAAATGTCTCATTCCGGTAACAATCCGATCGGCATGAGTCGAAAATAAATGGTTTGCGGTACATCTGGAAGCATTCCTCACAAACACGATCCAGACGTGCGAAGAAGGATGGGACAAATTCACCTTTACAGCCCAAUGGACCAGUAAAGAGCAGUCACAUAUCUAUUCCAUAGACUUGCUUCUUUGAAAUUUGGAUACACAGCUCCAAUCCAUGUCUUUCUUGGAUUAGGAUAUCUAUCUGCAAUCAGGUAGUUGAAACAUCCAGCACAAUGUAUAGCGAACAAGGGTTGTAAAGGTGAATTTGTTTTATTTTTTTTTGTATGTTTGGATTGTGTTTGTGAGGAATGTTTTTAGATGTATTGTAAATTATTTATTTTTGATTTATGTTGATTGGATTGTTATTGGAATGAGATATTTTTGTTGTGTATTGAAGTTTTAATGTTTTATAAGGAAAAGGATATGTTTGAAAGTATGGTAAATTAATTTTATGGATTTATTTTTTAAATTAGTAGGAGATTAATTGTGTTATAATTGTTATGAGTTTTTAGTTAAATAAATTGGTTATAGTAAATTAAGAGAATATTAATTGGATAAAATTTTTTTTAGTATTTAATATGAAAAAAAATTGTAATTTGAATGTTTTATTTAAATAAATTTATTATTTAGAAAATAGAAATAATATTTATGTTTATTTTTAAATGAAATTTAGGAAAAAATTAGGTTGGGTTGAAGTAAAGGTAATGTTGTTTATTTATGTATTTTTGTGAAGAAGTGGTTTTATTAAAAUGGACCAGUAAAGAGCAGUCACAUAUCUAUUCCAUAGACUUGCUUCUUUGAAAUUUGGAUACACAGCUCCAAUCCAUGUCUUUCUUGGAUUAGGAUAUCUAUCUGCAAUCAGGUAGUUGAAACAUCCAGCACAAUGUAUAGCGAACAATTAATAAAACCACTTCTTCGCAGAAGTGCATAAATAAACAGCGTTGCCTTTGCTTCAACCCAACCTAGTTTTTTCCTAAATTTCATTTAAAGGTAAACGTAGGTATTATTTCTATTTTCTAGATGGTGAATTTATTTGAGTGAAGCGTTCAAGTTACGGTTTTTTTTCGTATTAAATGCTAAAAGAAATTTTGTCCAGTTAATATTCTCTTGGTTTGCTGTAACCGATTTGTTTAGCTGAAGACTCGTAG | 1300 |  |
|  | Asymmetric | GGGUUGUGACGCAGUUGGUCUCCUGCUGAUUUAAGGGAUAGGUCCGUAGAGUUGAUUUACCAUACUUUCAAGCAUAUCCUUUUCCUUGUGGAGCAUUAGGGCUUCAGUGCACAACAAAAAUGUCUCAUUCCGGUAACAAUCCGAUCGGCAUGAGUCGAAAAUAAAUGGUUUGCGGUACAUCUGGAAGCAUUCCUCACAAACACGAUCCAGACGUGCGAAGAAGGAUGGGACAAAUUCACCUUUACAGCCCAAGGACCAGAAAGAGCAGCACAACACCAAGACGCCGAAAGGAACACAGCCCAACCAGCCGGAAGGAACACGCAACAGGAGGAAACACCAGCACAAGAAGCGAACAAGGGCUGUAAGGUGAAUUUGUCCCAUCCUCUUCGCACGUCUGGAUCGUGUUGUGAGGAAUGCUUCCAGAUGACCGCAAACCAUUUAUUUUCGCUCAUGCCGAUCGGAUUGUUACGGAAUGAGACAUUUUUGUUGGCACUGAAGCCCUAAUGCUCCCAAGGAAAAGGAUAUGCUUGAAGUAUGGUAAAUCAACUCUACGACCUAUCCCUUAAAUCAGCAGAGACCAACUGCGUCACAACCCUACGAGUCUUCAGCUAAACAAUCGGUUACAGCAAACCAAGAAAUAUUAACUGGACAAAAUUUUUUUAGCAUUUAAUACGAAAAAAACCGUAACUUGAACGCUUCCUCAAAUAAAUUCACCAUCUAAAAAUAGAAAUAAUACCUACGUUACCUUUAAAUGAAAUUUAGAAAAAACUAGGUUGGGUUGAACAAAGGCAACGCUGUUUAUUUUGCACUUCUGCGAAGAAGUGGUUUAUUAAAAGGACCAGAAAGAGCAGCACAACACCAAGACGCCGAAAGGAACACAGCCCAACCAGCCGGAAGGAACACGCAACAGGAGGAAACACCAGCACAAGAAGCGAACAAUUAAUAAAACCACUUCUUCGCAGAAGUGCAUAAAUAAACAGCGUUGCCUUUGCUUCAACCCAACCUAGUUUUUUCCUAAAUUUCAUUUAAAGGUAAACGUAGGUAUUAUUUCUAUUUUCUAGAUGGUGAAUUUAUUUGAGUGAAGCGUUCAAGUUACGGUUUUUUUUCGUAUUAAAUGCUAAAAGAAAUUUUGUCCAGUUAAUAUUCUCUUGGUUUGCUGUAACCGAUUUGUUUAGCUGAAGACUCGUAC | 1189 |  |

Supplementary Figure S 1: Mite mortality at 48 h following indirect dsRNA exposure Pero (Green) CHH (Purple). Parasitized adult bees were provided 50% sucrose solution containing 10 µg of ledGFP or the respective dsRNA constructs (conventional dsRNA, ledG:U, and led[Δ22]), alongside an untreated control. Mortality is expressed as the proportion of dead mites per treatment. Statistical comparisons against the control were performed using two-tailed Fisher’s exact tests, with significance defined as p < 0.05.

Supplementary Figure S 2: (A) Size distribution of siRNAs (18–30 nt) mapping to the modified GFP reference sequence. For each replicate, read counts for each length class and strand were normalized to the total number of mapped reads (sense and antisense combined). Bar heights represent the mean proportion of reads across biological replicates.(B) siRNA coverage profiles (18–30 nt) across the GFP sequence. Mean read depth represents the average coverage across three biological replicates (n = 3).
